# Silica Aerogel-supported Hydrozincite and Carbonate-intercalated Hydrotalcite for High-efficiency Removal of Pb(II) Ions by Precipitation Transformation Reactions

**DOI:** 10.1186/s11671-017-2323-2

**Published:** 2017-09-25

**Authors:** Lijun Wang, Xiaoxia Wang, Jianfa Li, Xiaolan Feng, Yusen Wang

**Affiliations:** School of Chemistry and Chemical Engineering, Shaoxing University, Shaoxing, 312000 People’s Republic of China

**Keywords:** Silica aerogel, Hydrotalcite, Adsorption, Lead removal

## Abstract

In this work, hydrozincite and Zn/Al-CO_3_^2−^ hydrotalcite supported on silica aerogel were prepared via a simple and economical process and used as adsorbents for Pb(II) removal. The supported hydrozincite and Zn/Al-CO_3_^2−^ hydrotalcite possess ultra-thin thickness, high surface area, and weak crystallinity. In the batch Pb(II) adsorption experiments, the adsorbents with higher Zn(II) contents showed higher Pb(II) adsorption capacities, and the adsorption data fitted well with the Langmuir isotherm model and pseudo-second-order kinetic model, indicating a mechanism of surface chemisorption. The adsorption capacities calculated based Langmuir isotherm model are 684.9 mg/g and 555.6 mg/g for the supported hydrozincite and Zn/Al-CO_3_^2−^ hydrotalcite, respectively, higher than the adsorption capacities of other hydrotalcite-based adsorbents and most of other inorganic adsorbents reported previously. The XRD diffraction peaks of hydrozincite and Zn/Al-CO_3_^2−^ hydrotalcite disappeared after the adsorption, and the Pb(II) species were uniformly dispersed in the adsorbents in form of Pb_3_(CO_3_)_2_(OH)_2_ proven by TEM, EDS mapping and XRD analysis, demonstrating the nature of the adsorption is the precipitation conversion of hydrozincite or Zn/Al-CO_3_^2−^ hydrotalcite into Pb_3_(CO_3_)_2_(OH)_2_. These results demonstrate the synergic Pb(II) removal effect of the CO_3_^2−^ and OH^−^ derived from hydrozincite and Zn/Al-CO_3_^2−^ hydrotalcite together with their ultra-thin thickness and high surface area contribute the excellent properties of the adsorbents.

**Electronic supplementary material:**

The online version of this article (10.1186/s11671-017-2323-2) contains supplementary material, which is available to authorized users.

## Background

Heavy metal (e.g., Pb, Cd, and Hg) pollution in water distribution systems and surface water causes serious environmental and living organism health problems and has been a major global concern for many years [1, 2]. Various technologies including chemical precipitation [3], adsorption [4, 5], ion-exchange [6, 7], etc. have been used for removal of high toxic heavy metal ions from water, among which adsorption is the most extensively used one because of its operation simplicity, high efficiency [8, 9], and little influence on survival environment of aquatic biology such as pH [10], for which there would be large fluctuation when removing heavy metal ions by chemical precipitation using high concentrations of precipitant reagents. For these reasons, a number of absorbent materials such as inorganic materials [11–15], polymers [16], biomaterials [17, 18], and sorption resins [7, 8], have been developed and applied for adsorption of toxic metal ions from wastewater. However, there are still some challenges that restrict the adsorption approach, such as limited surface area and corresponding low adsorption capacities for most of the adsorbents. Hence, it is desirable to explore novel high surface area adsorbent materials for the removal of heavy metal ions with high efficiency.

Hydrotalcites (HTs, also called layered double hydroxides), consisting of stacked brucite type octahedral layers which are composed of bivalent and trivalent metal hydroxides with anions and water molecules occupying the interlayer space, have been extensively used for adsorbing various anions by ion-exchange [19–21]. These materials were also studied as sorbents and scavengers of heavy metal cations from waters in recent years [22–26]. Low-cost, simple preparation, and high sorption efficiency make these materials suitable for the application in the field of wastewater treatment [27, 28]. HTs themselves could adsorb toxic metal cations through three routes: (1) isomorphous substitution of divalent metal ions in HT crystal by toxic divalent metal ions with similar ionic radiuses in the solution [24, 27]; (2) reaction of metal hydroxide (mainly divalent metal hydroxides) component in HTs with toxic metal ions with different ionic radiuses [29]; (3) reaction of interlayer carbonate with toxic metal ions [29]. From the above, interlayer carbonate and divalent metal hydroxide components in the layers are main functional components in HTs for the toxic metal ion adsorption, and thereby carbonate-intercalated HTs (carbonate-HTs) containing both components are expected to have optimum adsorption performance of metal ions. However, HT powders synthesized by conventional methods typically have low specific surface areas [30], which restrict their adsorption properties. A good way to improve the surface area of HTs is to support them on carriers with high surface area. Li et al. [31] prepared porous materials (SBA-15) supported HTs with high surface area by long-time hydrothermal treatment of the pre-prepared SBA-15 supported mixed metal oxides. Jong et al. [32] synthesized carbon nanofibers supported HT platelets with small size and enhanced surface area. However, the above supporting methods are somewhat complicated, and the support materials are expensive. Moreover, there are few reports on adsorption of toxic metal ions by carbonate hydroxide salts of divalent metal, which also contain both carbonate and divalent metal hydroxides like carbonate-HTs. Therefore, developing low-cost and easy-prepared supported carbonate hydroxide salts and carbonate-HTs with high surface area is expected to enhance the adsorption of toxic metal ions.

Silica aerogels (SAs) have drawn a lot of interest both in science and technology because of their low bulk density, high surface area, and low thermal conductivity [33, 34]. Furthermore, SA is inexpensive (US $2 ~ 3/Kg) due to its fabrication on a massive scale and very large demand in thermal insulation industry. Therefore, in the present work, hydrozincite (Zn_5_(OH)_6_(CO_3_)_2_) and Zn/Al-CO_3_^2−^ hydrotalcite supported on commercial SA, which provides confined space and nucleation sites for loading and growth of hydrozincite and Zn/Al-CO_3_^2−^ hydrotalcite, were prepared by a facile method. The texture properties of the SA supported hydrozincite and Zn/Al-CO_3_^2−^ hydrotalcite were characterized by TEM, XRD, ICP, and BET. The impact of Zn(II) ion contents and pH for synthesis of adsorbents on the adsorption capacities was especially evaluated. In addition, the adsorption equilibrium and kinetics were investigated and fitted with the corresponding isotherm models and kinetics models, respectively. The adsorbents after the adsorption were characterized by TEM, XRD, and EDS mapping. Finally, a possible adsorption mechanism in this adsorption system was discussed.

## Methods

### Materials

Zinc nitrate, aluminum nitrate, sodium hydrogen carbonate, sodium hydroxide, and lead nitrate were all of analytical grade and purchased from Aladdin Reagent Co., Ltd. (Shanghai, China). Hydrochloric acid (36–38%) was of analytical grade and purchased from Sinopharm Chemical Reagent Co., China (Shanghai, China). SA was provided by Nano Tech Co, LTD (Shaoxing, China).

### Preparation of the adsorbents

The SA powder was calcined at 823 K for 2 h to remove the organic groups on the surface before use. The treated SA (500 mg) was dispersed in 500 mL of deionized water by ultrasound for 30 min. To the obtained SA suspension, 25 mL mixed solution (A) of *m* mol Zn(NO_3_)_2_ and *n* mol Al(NO_3_)_3_ in 150 mL of deionized water was added and stirred for 5 min, and then 125 mL mixed solution A and another mixed solution of NaOH/NaHCO_3_ (0.5 M/0.5 M) was added dropwise alternately. The Zn:Al ratio was varied such that *m* + *n* = 0.0075 mol and *m*:*n* = 3:0, 3:1, 2:1, and 0:1. The final pH values of the solutions were adjusted to 8.8 or 9.5 with the above mixed solution of NaOH/NaHCO_3_ followed by hydrothermal treatment at 80 °C for 24 h. The obtained products were collected by centrifugation, washed with deionized water three times, and vacuum-dried. The final samples of SA supported hydrozincite (hydrozincite, *m*:*n* = 3:0), and Zn/Al hydrotalcite (*m*:*n* = 3:1 and 2:1) were denoted as SA-Zn-HZ and SA-Zn_*x*_Al-HT, respectively, where *x* represents divalent metal/trivalent metal mole ratio in the precursor solution. The control sample of SA supported aluminum hydroxide was designated as SA-Al-H (*m*:*n* = 0:1).

### Characterization

Transmission electron micrographs were taken using the JEM-1011 electron microscope operating at an accelerating voltage of 80 kV. Scanning electron micrographs and energy-dispersive spectra were obtained using the JSM-6360LV scanning electron microscope equipped with an X-act energy-dispersive X-ray (EDX) analyzer (Oxford INCA). N_2_ adsorption-desorption isotherms were obtained using a Micromeritics ASAP TriStar II 3020 pore analyzer at 77 K under continuous adsorption conditions. The samples were outgassed at 150 °C for 8 h before measurements. The specific surface areas were calculated by the Brunauer-Emmett-Teller method, and pore size distributions were measured using Barrett-Joyner-Halenda analysis from the desorption branches of nitrogen isotherms. X-ray diffraction (XRD) pattern was collected using an Empyrean XRD diffractometer. The element contents of Zn, Si, and Al of the adsorbents were determined using inductively coupled plasma atomic emission spectroscopy (Leeman Prodigy XP ICP-AES spectrometer).

### Adsorption Experiment

The Pb(II) aqueous solution (1000 ppm) was prepared by dissolving Pb(NO_3_)_2_ in deionized water. Afterwards, it was diluted with deionized water to a desired concentration. The Pb(II) solutions with different concentrations were all adjusted to achieve pH at about 6.0 with 0.1 mol L^−1^ HCl or NaOH aqueous solutions. Typically, 50 mg of adsorbents were placed into 100 mL Pb(II) aqueous solutions with different concentrations (100, 200, 300, 400, 500, and 1000 ppm) in Erlenmeyer flask, respectively. Then, the Erlenmeyer flask was shaken (150 rpm) in an incubator shaker with a set temperature of 25 °C for a period of 24 h to reach adsorption equilibrium. At the end of each adsorption process, the suspensions were centrifuged and the supernatants were filtered and used to determine the amounts of Pb(II) by Shimadzu AA-6300 atomic adsorption spectrophotometer (AAS). The adsorption capacities of the adsorbents were calculated according to the following equation: *q*_e_ = (*C*_0_ − *C*_e_)V/m, where *q*_*e*_ represents the adsorbed amount at equilibrium (mg g^−1^), *C*_*0*_ and *C*_*e*_ are the initial and equilibrium concentrations of pollutants in solution (mg L^−1^), V is the volume of the Pb(II) solutions (mL), and m is the dry weight of adsorbents (g). For the adsorption kinetics analysis, 50 mg of adsorbents were added into 100 mL Pb(II) solution with a concentration of 500 ppm. The suspensions were shaken (150 rpm) at 25 °C. At specific reaction time intervals of 10, 30, 50, 70, 100, 140, 180, 240, 360, 600, and 1440 min, 2 mL of suspensions were taken and filtered by 0.22 mL membrane. The Pb(II) concentration in the filtrate was analyzed by flame atomic adsorption spectrophotometer (Shimadzu AA-6300).

## Results and Discussion

### The Optimization of Synthesis Parameters

**Fig. 1:**
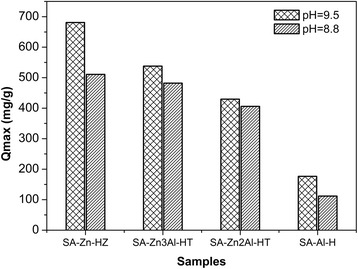
The maximum adsorption capacities of SA-Zn-HZ, SA-Zn_*x*_Al-HT, and the control sample SA-Al-H with various Zn contents. From the left to the right, the Zn element contents decreased stepwise

In order to investigate the effect of synthesis parameters of the adsorbents on their adsorption performance, the maximum adsorption capacities of SA-Zn-HZ, SA-Zn_3_Al-HT, SA-Zn_2_Al-HT, and SA-Al-H prepared under varied Zn/Al precursor ratios (3:0, 3:1, 2:1, and 0:1) and pH (8.8 and 9.5) were tested (Fig. 1). The maximum adsorption capacities are 680.8 mg g^−1^, 537.8 mg g^−1^, 429.5 mg g^−1^, and 176.4 mg g^−1^, respectively, for SA-Zn-HZ, SA-Zn_3_Al-HT, SA-Zn_2_Al-HT, and SA-Al-H prepared at pH of 9.5, and those are 510.6, 482.2, 405.7, and 111.8 mg/g, respectively, for SA-Zn-HZ, SA-Zn_3_Al-HT, SA-Zn_2_Al-HT, and SA-Al-H prepared at pH of 8.8. Therefore, it can be concluded that high divalent metal contents and pH (such as 9.5) on synthetic medium are beneficial to the promotion of adsorption properties. The SA-Zn-HZ and SA-Zn_3_Al-HT synthesized at pH of 9.5 with high adsorption capacities were taken for the textural characters, adsorption equilibrium, and kinetics research in the following sections.

### The Texture Characters of SA-Zn-HZ and SA-Zn_3_Al-HT

**Fig. 2:**
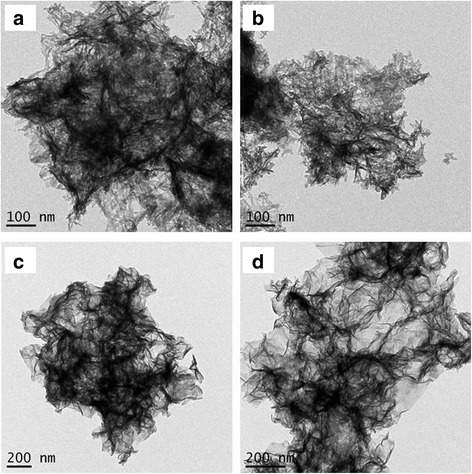
TEM images of SA-Zn-HZ (**a**, **b**), SA-Zn_3_Al-HT (**c**, **d**) prepared at pH of 9.5

**Fig. 3:**
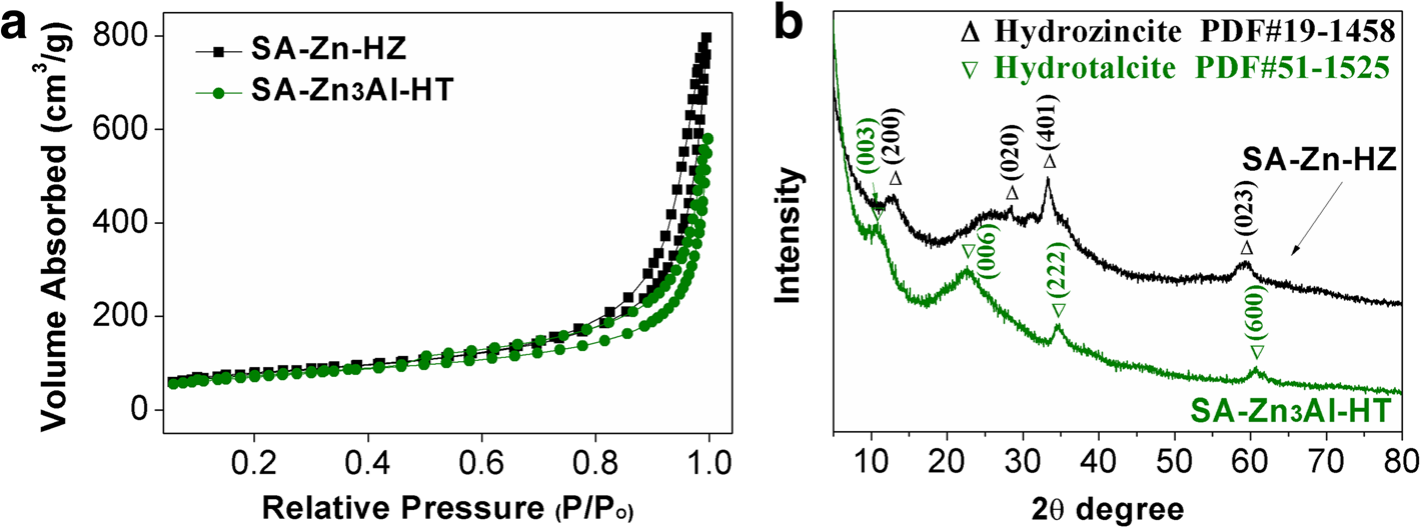
N_2_ adsorption-desorption isotherms (**a**) and XRD patterns (**b**) of SA-Zn-HZ and SA-Zn_3_Al-HT

The morphology of SA-Zn-HZ, SA-Zn_3_Al-HT, and the carrier SA was characterized by TEM as shown in Fig. 2 and Additional file 1: Figure S1. Hydrozincite (HZ) and Zn/Al-CO_3_^2−^ HT in SA-Zn-HZ and SA-Zn_3_Al-HT composites, respectively, all showed flake structure with ultra-thin thickness (< 5 nm). The textural parameters of SA-Zn-HZ and SA-Zn_3_Al-HT are listed in Table 1, and the surface areas of SA-Zn-HZ and SA-Zn_3_Al-HT calculated from N_2_ adsorption-desorption isotherms (Fig. 3a) are 264.1 m^2^ g^−1^ and 233.9 m^2^ g^−1^, respectively, lower than that of the substrate SA (Additional file 1: Table S1 and Figure S2), due to the possible pore structure blocking of SA by lamellar HZ and Zn/Al-CO_3_^2−^ HT and the higher density of HZ and HT than that of SA. As shown in Fig. 3b, the XRD patterns present a hydrozincite structure (PDF#19-1458, Zn_5_(CO_3_)_2_(OH)_6_) for SA-Zn-HZ and a typical carbonate-intercalated hydrotalcite structure (PDF#51-1525) for SA-Zn_3_Al-HT, respectively. However, the strength of characteristic diffraction peaks of the both samples is weak indicating the ultra-thin layered structure and the relative weak crystallinity of HZ and HT in the composites.

**Table 1:** The textural parameters of SA-Zn-HZ and SA-Zn_3_Al-HT

Samples	Zn content^a^	Al content^a^	Si content^a^	*S*_BET_ m^2^ g^−1^	*V*_*p*_ cm^3^ g^−1^	*D*_*p*_^b^ nm
SA-Zn-HZ	46.30%	0	53.70%	264.1	0.94	21.1
SA-Zn_3_Al-HT	39.0%	16.90%	44.10%	233.9	0.75	16.1

^a^Obtained via ICP-OES measurement

^b^Calculated from desorption branch of nitrogen isotherms

### Adsorption Isotherms of Pb(II) Ions on SA-Zn-HZ and SA-Zn_3_Al-HT

**Fig. 4:**
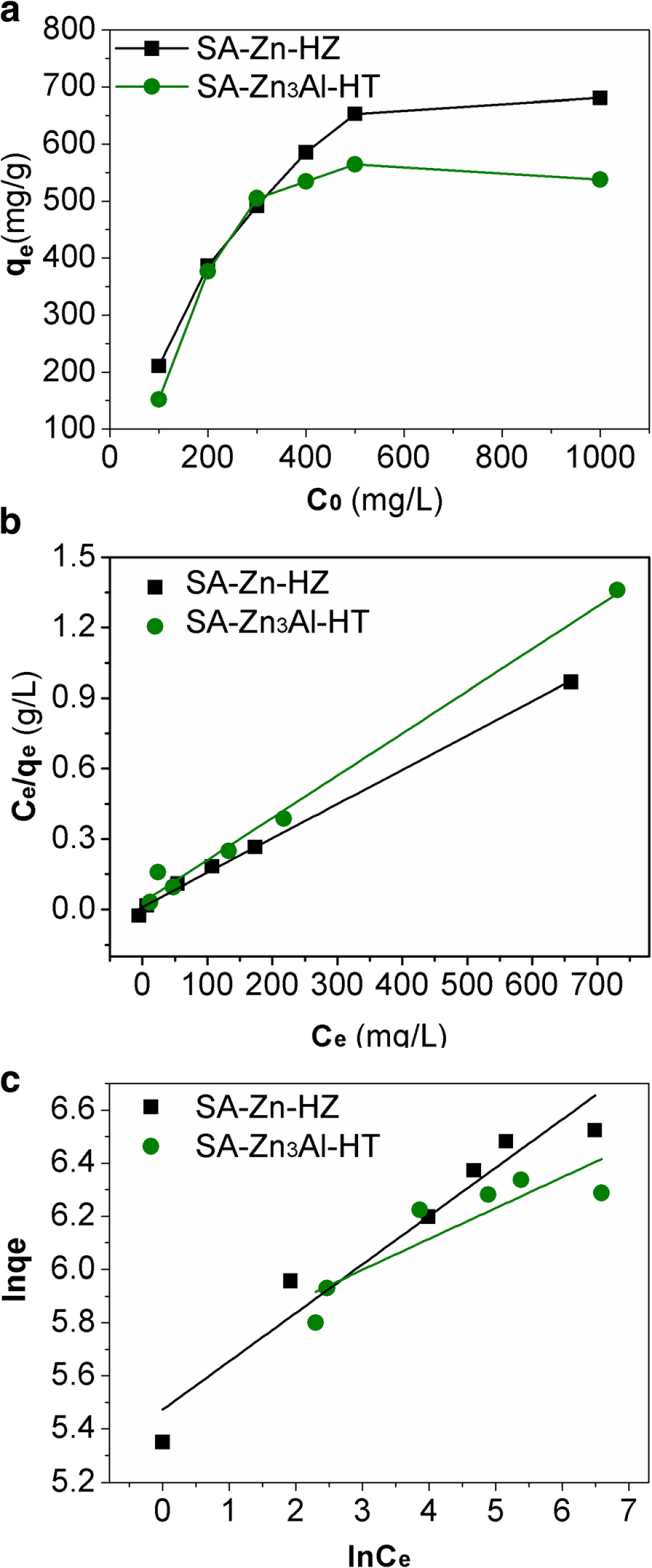
Adsorption isotherms of Pb(II) on SA-Zn-HZ and SA-Zn_3_Al-HT (**a**), Langmuir (**b**), and Freundlich (**c**) adsorption isotherm models fitting for Pb(II) adsorption. Experiment conditions: initial Pb(II) concentration 100 ~ 1000 ppm; adsorbent dose 0.5 g L^−1^; contact time 24 h, solution pH 6.0 ± 0.1; temperature 30 °C

The effect of initial Pb(II) concentrations (100, 200, 300, 400, 500, and 1000 ppm) on adsorption properties of SA-Zn-HZ and SA-Zn_3_Al-HT is included in Fig. 4a. As expected, the adsorption capacities for both adsorbents increased with the ascending initial concentrations of Pb(II). At low initial concentrations of 100, 200, and 300 ppm, the adsorption capacities for Pb(II) were nearly linearly proportional to the initial Pb(II) concentrations for both SA-Zn-HZ and SA-Zn_3_Al-HT. However, at high initial Pb(II) concentrations of 400, 500, and 1000 ppm, the adsorption capacities increase slowly and nearly approach their maximum adsorption capacities, which is due to the lack of available adsorption sites on adsorbents that can accommodate more Pb(II) ions. The adsorption isotherm data was fitted with the Langmuir, Freundlich, Sips, and Redlich–Peterson models which were represented mathematically as Eqs. (1), (2), (3), and (4) [13, 24, 35–37], respectively:

1 $$ {C}_e/{q}_e=1/\left({q}_m{K}_L\right)+{C}_e/{q}_m $$

2 $$ \ln {q}_e=\ln {K}_F+\left(1/n\right)\ln {C}_e $$

3 $$ {q}_e={q}_m{\left({K}_S{C}_e\right)}^{n{}_s}/\left\{1+{\left({K}_S{C}_e\right)}^{n{}_s}\right\} $$

4 $$ {q}_e={q}_m\left({K}_{\mathrm{RP}}{C}_e\right)/\left\{1+{\left({K}_{\mathrm{RP}}{C}_e\right)}^{n_{RP}}\right\} $$

Where *C*_e_ (mg/L) is the equilibrium concentration in the aqueous phase; *q*_e_ (mg/g) is the equilibrium amount adsorbed by the adsorbent; *q*_m_ (mg/g) denotes the theoretical saturated adsorption capacity; *K*_L_ (L/mg) is Langmuir constant related to the adsorption-desorption energy and the affinity of binding sites for ions; *K*_*F*_ is roughly an indicator of the adsorption capacity, and 1/*n* is the adsorption intensity; *K*_*S*_ (L/mmol) is the Sips isotherm constant and *n*_*S*_ is the Freundlich heterogeneity factor; *K*_RP_ (L/mg) and *n*_RP_ are Redlich–Peterson constants. The adsorption data and fitting plots of Langmuir, Freundlich, Sips and Redlich-Peterson models for Pb(II) adsorption on SA-Zn-HZ and SA-Zn_3_Al-HT were shown in Fig. 4b, c and Additional file 1: Figure S3, and the calculated parameters of these models were given in Table 2 and Additional file 1: Table S2. For the four studied models, the Langmuir model showed more significant correlation coefficients (*R* ^2^ ≥ 0.99) with the experimental data than those of Freundlich, Sips and Redlich-Peterson models, respectively, and the maximum adsorption capacities of the SA-Zn-HZ and SA-Zn_3_Al-HT for Pb(II) based on Langmuir model are 684.9 mg/g and 555.6 mg/g, respectively.

**Table 2:** The parameters of the Langmuir and Freundlich models fitted to the experimental data of Pb(II) adsorption on SA-Zn-HZ and SA-Zn_3_Al-HT

Adsorbent	*q*_*e*, exp_ (mg g^−1^)	Langmuir model parameters			Freundlich model parameters		
		*q*_*m*_ (mg g^−1^)	*k*_L_ (L mg^−1^)	*R* ^2^	*n*	*k*_*F*_ ((mg g^−1^)(L mg^−1^)^1/n^)	*R* ^2^
SA-Zn-HZ	680.8	684.9	0.131	99.7%	5.50	238.2	92.6%
SA- Zn_3_Al-HT	537.8	555.6	0.059	99.0%	11.36	331.5	62.3%

### Adsorption Kinetics of Pb(II) Ions on SA-Zn-HZ and SA-Zn_3_Al-HT

**Fig. 5:**
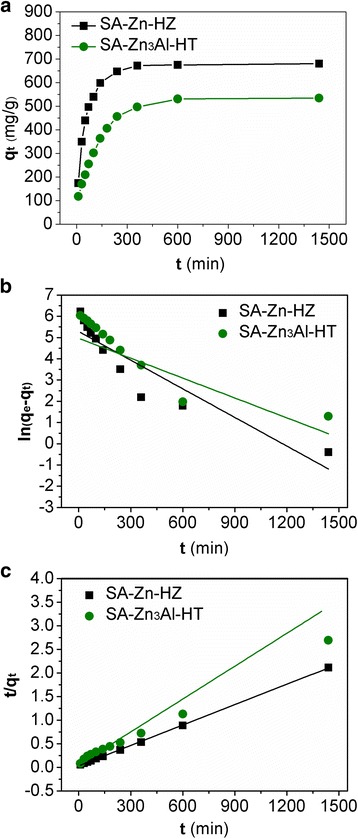
Adsorption kinetics of Pb**(**II**)** on SA-Zn-HZ and SA-Zn_3_Al-HT (**a**) and plots of the pseudo-first-order (**b**) and pseudo-second-order (**c**) adsorption kinetic models fitted for Pb(II) adsorption. Experiment conditions: initial Pb(II) concentration 500 ppm; adsorbent dose 0.5 g L^−1^; contact time 0 ~ 24 h, solution pH 6.0 ± 0.1; temperature 30 °C

Adsorption kinetics was investigated to determine the time required for adsorption equilibrium and explain the Pb(II) adsorption mechanism on the adsorbents. Adsorption kinetic data of Pb(II) on SA-Zn-HZ and SA-Zn_3_Al-HT from 0 to 1440 min (24 h) was presented in Fig. 5a. It can be seen that the adsorption rates for both adsorbents were rapid within the first 50 min, then gradually slowed down, and thereafter, the adsorption equilibrium was reached. The fast removal rates of Pb(II) in the beginning may be attributed to the rapid diffusion of Pb(II) from the bulk solution to the external surfaces of the adsorbents and a large amount of available sites of the adsorbents at the initial stage. At later stages, the slow adsorption process was probably attributed to the longer diffusion distance of Pb(II) onto the adsorbent and limited surface adsorption sites of the adsorbents. To investigate the adsorption mechanism of Pb(II) on SA-Zn-HZ and SA-Zn_3_Al-HT, the experimental results were fitted with the pseudo-first-order and pseudo-second-order kinetic models as expressed by Eqs. (5) and (6) [35, 38], respectively.

5 $$ \ln \left({q}_e-{q}_t\right)=\ln {q}_e-{k}_1t $$

6 $$ t/{q}_t=1/\left({k}_2{q_e}^2\right)+t/{q}_e $$

where *q*_t_ (mg/g) is the amount of the adsorbate removed by the adsorbent at time *t* (min); *q*_e_ (mg/g) is the equilibrium adsorption capacity; *k*_1_ (min^−1^) and *k*_2_ (g/(mg min^−1^)) are the rate constants of pseudo-first-order and pseudo-second-order models, respectively. The linear plots of the ln(*q*_e_-*q*_t_) versus *t* and the plots of *t*/*q*_t_ against *t* for the pseudo-first-order and pseudo-second-order kinetic models, respectively, are shown in Fig. 5b, c. The adsorption capacities (*q*_e_) and the rate constants (*k*_1_ and *k*_*2*_) calculated from the slopes and intercepts of the plots are summarized in Table 3. Obviously, the pseudo-second-order model with higher correlation coefficients (*R* ^2^ > 0.99) can fit the experimental kinetic data better than the pseudo-first-order model. In addition, the values of adsorption capacity (*q*_*e*, cal_) calculated from the pseudo-second-order model are very consistent with the experimental *q*_e_ values (*q*_*e,* exp_). These results suggested that the pseudo-second-order kinetic model sorption mechanism was predominant, demonstrating that the overall rate of the Pb(II) uptake appeared to be controlled by a chemisorption process [13, 39].

**Table 3:** The pseudo-first-order and pseudo-second-order kinetic model parameters for the adsorption of Pb(II) on SA-Zn-HZ and SA-Zn_3_Al-HT

Adsorbent	*q*_*e*,exp_ (mg/g)	Pseudo-first-order model			Pseudo-second-order model		
		*k*_1_ × 10^3^ (min^−1^)	*q*_*e*_ (mg/g)	*R* ^2^	*k*_2_ × 10^3^ (g/mg min)	*q*_*e,*cal_ (mg/g)	*R* ^2^
SA-Zn-HZ	680.8	4.50	197.4	84.4%	0.058	694.4	99.9%
SA-Zn_3_Al-HT	537.8	3.55	273.2	82.8%	0.026	565.0	99.7%

### Adsorption Mechanism and Performance Evaluation

**Fig. 6:**
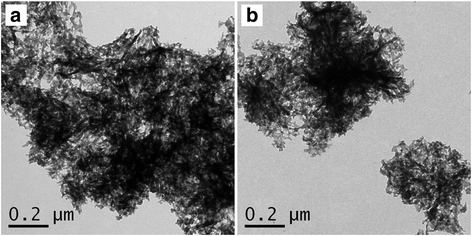
TEM images of SA-Zn-HZ-Pb (**a**) and SA-Zn_3_Al-HT-Pb (**b**)

**Fig. 7:**
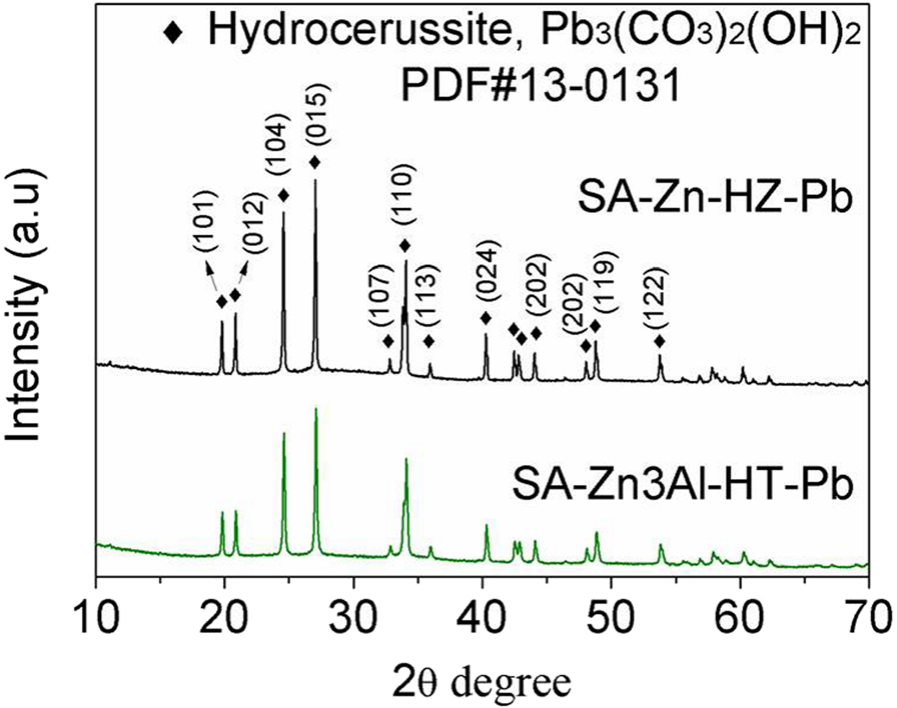
XRD patterns of SA-Zn-HZ-Pb and SA-Zn_3_Al-HT-Pb

**Fig. 8:**
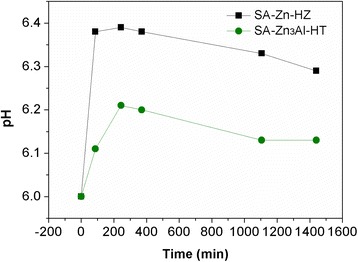
pH values of the Pb(II) solutions versus time in the adsorption using SA-Zn-HZ and SA-Zn_3_Al-HT as adsorbents

To further explore the mechanism of Pb(II) adsorption on SA-Zn-HZ and SA-Zn_3_Al-HT, the samples SA-Zn-HZ and SA-Zn_3_Al-HT after adsorption (SA-Zn-HZ-Pb and SA-Zn_3_Al-HT-Pb) were characterized by TEM, EDS mapping, and XRD. Both SA-Zn-HZ-Pb and SA-Zn_3_Al-HT-Pb (Fig. 6) have higher contrast than SA-Zn-HZ and SA-Zn_3_Al-HT in TEM images (Fig. 2), respectively, demonstrating high atomic number of Pb has been adsorbed onto the adsorbents from the solution. In EDS elemental mapping of Additional file 1: Figure S4 and Figure S5, it is obvious that the Pb element is uniformly dispersed in the SA-Zn-HZ-Pb and SA-Zn_3_Al-HT-Pb, indicating indirectly the heterogeneous nucleation of Pb(II) species on the surface of adsorbents. The XRD analysis (Fig. 7) showed the Pb(II) species in SA-Zn-HZ-Pb and SA-Zn_3_Al-HT-Pb all exist in form of Pb_3_(CO_3_)_2_(OH)_2_ (Hydrocerussite, PDF#13-0131), which is more stable than Pb(OH)_2_ or PbCO_3_ owing to the lower solubility product constant of the former (3.16 × 10^−46^) compared with those of the latter (1.43 × 10^−15^ for Pb(OH)_2_ and 7.9 × 10^−14^ for PbCO_3_) [40, 41]. The solution pH changes over time in the adsorption are presented in Fig. 8. At the initial contact time (0 ~ 210 min), the solution pH continuously increased mainly because the hydrozincite and hydrotalcite composed of hydroxides were dissolved to reach a precipitation-dissolution equilibrium in the weakly acidic Pb solution which resulted in the release of OH^−^. However, the solution pH gradually decreased with continue increase of contact time (210 ~ 1440 min), which is probably because the produced hydrocerussite in the adsorption deposited on the surface of hydrozincite and hydrotalcite prevented the further dissolution of the both adsorbents and the subsequent release of OH^−^, meanwhile the OH^−^ was sequentially consumed to generate hydrocerussite together with CO_3_^2−^ and Pb^2+^ in the solution. Overall, the Pb(II) solutions has a low pH fluctuation with a range of 6.0 ~ 6.39 and 6.0 ~ 6.21 in the adsorption process using SA-Zn-HZ and SA-Zn_3_Al-HT as adsorbents, respectively, manifesting both the adsorbents have low impact on pH of the water body.

**Fig. 9:**
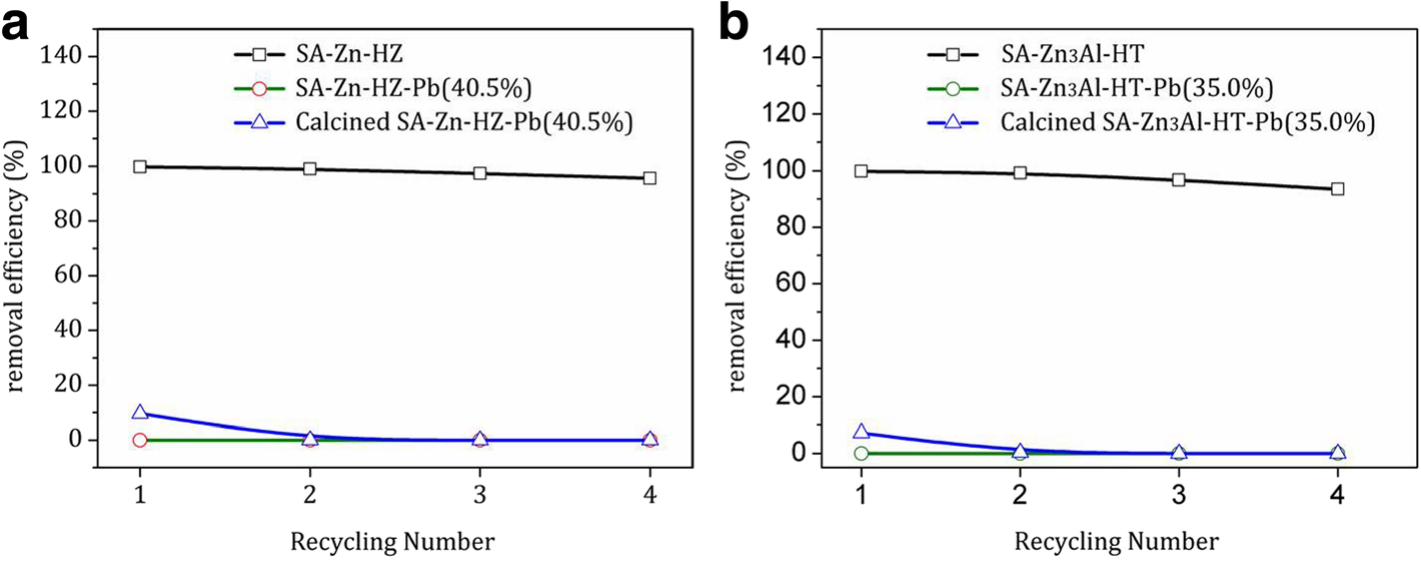
Recycling for the removal of Pb(II) by **a** pristine SA-Zn-HZ, saturated SA-Zn-HZ (SA-Zn-HZ-Pb (40.5%)), and saturated SA-Zn-HZ after calcination; **b** pristine SA-Zn_3_Al-HT, saturated SA-Zn_3_Al-HT (SA-Zn-HZ-Pb (35.0%)), and saturated SA-Zn_3_Al-HT after calcination. Initial Pb(II) concentration 100 ppm; adsorbent dose 0.5 g L^−1^; contact time 24 h, solution pH 6.0 ± 0.1; temperature 30 °C

In this adsorption system, the isomorphic substitution of divalent of Zn(II) in SA-Zn-HZ and SA-Zn_3_Al-HT by Pb(II) is impossible because of the much larger ionic radius of Pb(II) (0.119 nm) than that of Zn(II) (0.074 nm). Therefore, the reaction of HZ or Zn/Al-CO_3_^2−^ HT with toxic Pb(II) cations probably made major contribution for the adsorption, and resulted in the precipitation of hydrocerussite. The precipitation transformation from HZ (or Zn/Al-CO_3_^2−^ HT) to hydrocerussite in the Pb(II)-containing aqueous solution was probably due to the lower solubility product constant of the later than that of the former [42]. In addition, in this adsorption system, the adsorption process based on the surface precipitation transformation reactions is irreversible. Before reaching the saturated adsorption, the catalysts can be reused repeatedly and showed high removal efficiency (> 93.5%) in each cycle (Fig. 9). Once the saturated adsorption is reached, the adsorbents cannot be reused even after calcination, which can be attributed to the reason that the surface functional components (hydrozincite and hydrotalcite) in the saturated adsorbents have transformed into hydrocerussite and further adsorption is restricted.

The maximum adsorption capacities of the SA-Zn-HZ and SA-Zn_3_Al-HT for Pb(II) based on Langmuir isotherm model are 684.9 and 555.6 mg/g, respectively, higher than those of other hydrotalcite-based adsorbents and most of other inorganic adsorbents such as the graphene oxide, carbon nanotubes, and activated carbon-based adsorbents (Table 4). The superior adsorption properties together with the low-cost and the ease of preparation make the SA-Zn-HZ and SA-Zn_3_Al-HT highly competitive scavengers for Pb(II) removal from the wastewater.

**Table 4:** Comparison of Pb(II) adsorption capacities of SA-Zn-HZ and SA-Zn_3_Al-HT with other inorganic adsorbents

Adsorbents	*Q*_max_^a^ (mg g^−1^)	Reference	Adsorbents	*Q*_max_^a^ (mg g^−1^)	Reference
SA-Zn-HZ	684.9	This work	Fe_3_O_4_@Zr(OH)_x_	310.8	[43]
SA-Zn_3_Al-HT	555.6	This work	GO (19.2 kGy)	636.4	[44]
Mg_2_Al-LS-HT	142.8	[35]	EDTA-modified GO	479.0	[45]
Fe_3_O_4_/GO/Mg_3_Al-HT	173.0	[24]	Graphene aerogel	373.8	[46]
MnO_2_-HT	49.9	[47]	Activated carbon	26.5	[48]
HT-Cl	250.5	[27]	MgFe_2_O_4_	113.7	[49]
CoMo HT	73.4	[50]	Fe_2_O_3_@AlOOH	84.1	[51]
Al(OH)_3_/PAA-co-PAM	106.2	[52]	Fe_3_O_4_@SiO_2_-EDTA	114.9	[53]
NC/FeMg HT	344.8	[1]	CNTs/CoFe_2_O_4_	140.1	[54]
*g*-C_3_N_4_	65.6	[13]	C nanofibers	795.7	[15]
WO_x_/C	1224.7	[14]	MoS_2_/rGO	412.8	[12]

^a^Calculated from the Langmuir isotherm model for Pb(II) adsorption onto adsorbents

## Conclusions

We have demonstrated the textural properties, Pb(II) adsorption properties, adsorption kinetics, and possible adsorption mechanism of silica aerogel supported hydrozincite and Zn-Al-CO_3_^2−^ hydrotalcite. The both supported hydrozincite and Zn/Al-CO_3_^2−^ hydrotalcite possess ultra-thin thickness (< 5 nm) and high surface area. In the batch Pb(II) adsorption experiments, the adsorption data fitted well with the Langmuir isotherm model and pseudo-second-order kinetic model, indicating a surface chemisorption process. The saturated adsorption capacities calculated based on Langmuir isotherm model are 684.9 and 555.6 mg/g for the supported hydrozincite and Zn/Al-CO_3_^2−^ hydrotalcite, respectively, close to the experiment values and higher than the adsorption capacities of other hydrotalcite-based adsorbents and most of other inorganic adsorbents reported previously. After the adsorption, the Pb(II) species adsorbed on the adsorbents exist in form of hydrocerussite and the XRD diffraction peaks of hydrozincite or Zn/Al-CO_3_^2−^ hydrotalcite disappeared, demonstrating the nature of the adsorption is probably the precipitation conversion of hydrozincite or Zn/Al-CO_3_^2−^ hydrotalcite into hydrocerussite with a low solubility product constant in the Pb(II) solution. Finally, this work would provide a reference for developing novel heavy metal ion absorbents, e.g.*,* immobilization toxic metal ions on the surface of the adsorbents in form of special precipitates with low solubility product constants by precipitation transformation reactions.

## Additional file

Additional file 1: Figure S1. TEM images of silica aerogel with disordered pores. **Figure S2.** N_2_ adsorption − desorption isotherms and the corresponding BJH pore size distribution of SA. **Table S1.** BET surface area (*S*_BET_), pore volume (*V*_*p*_) and average pore diameter of SA. **Figure S3.** Sips (a) and Redlich–Peterson (b) adsorption isotherm models fitting for Pb(II) adsorption. Initial Pb(II) concentration 100 ~ 1000 ppm; adsorbent dose 0.5 g L^−1^; contact time 24 h, solution pH 6.0 ± 0.1; temperature 30 °C. **Table S2.** The parameters of the Sips and Redlich–Peterson models fitted to the experimental data of Pb(II) adsorption on SA-Zn-HZ and SA-Zn_3_Al-HT. **Figure S4.** EDS elemental mapping and the corresponding elemental analysis reports of the SA-Zn-HZ-Pb after the adsorption. **Figure S5.** EDS elemental mapping and the corresponding elemental analysis reports of the SA-Zn_3_Al-HT-Pb after the adsorption. (DOCX 3589 kb)
