# Supplementary material for: Silica Aerogel-supported Hydrozincite and Carbonate-intercalated Hydrotalcite for High-efficiency Removal of Pb(II) Ions by Precipitation Transformation Reactions
Source: Nanoscale Res Lett. 2017 Sep 25;12:549. doi: 10.1186/s11671-017-2323-2 (PMC5612910; doi:10.1186/s11671-017-2323-2)
Supplement: Additional file 1: Figure S1. — TEM images of silica aerogel with disordered pores. Figure S2. N2 adsorption − desorption isotherms and the corresponding BJH pore size distribution of SA. Table S1. BET surface area (S BET), pore volume (V p) and average pore diameter of SA. Figure S3. Sips (a) and Redlich–Peterson (b) adsorption isotherm models fitting for Pb(II) adsorption. Initial Pb(II) concentration 100 ~ 1000 ppm; adsorbent dose 0.5 g L−1; contact time 24 h, solution pH 6.0 ± 0.1; temperature 30 °C. Table S2. The parameters of the Sips and Redlich–Peterson models fitted to the experimental data of Pb(II) adsorption on SA-Zn-HZ and SA-Zn3Al-HT. Figure S4. EDS elemental mapping and the corresponding elemental analysis reports of the SA-Zn-HZ-Pb after the adsorption. Figure S5. EDS elemental mapping and the corresponding elemental analysis reports of the SA-Zn3Al-HT-Pb after the adsorption. (DOCX 3589 kb) [file 11671_2017_2323_MOESM1_ESM.docx]

# *Additional file 1*

**Silica aerogel supported** **hydrozincite and carbonate-intercalated hydrotalcite for high-efficiency removal of Pb(II) ions by precipitation transformation reactions**

Lijun Wang^1,^*, Xiaoxia Wang^1^, Jianfa Li^1^, Xiaolan Feng^1^, Yusen Wang^1^

*^1^School of Chemistry and Chemical Engineering, Shaoxing University, Shaoxing 312000, PR China*

**Corresponding author. E-mail address: ljwang@usx.edu.cn*

**Figures and Tables**







**Figure S1** TEM images of silica aerogel with disordered pores.


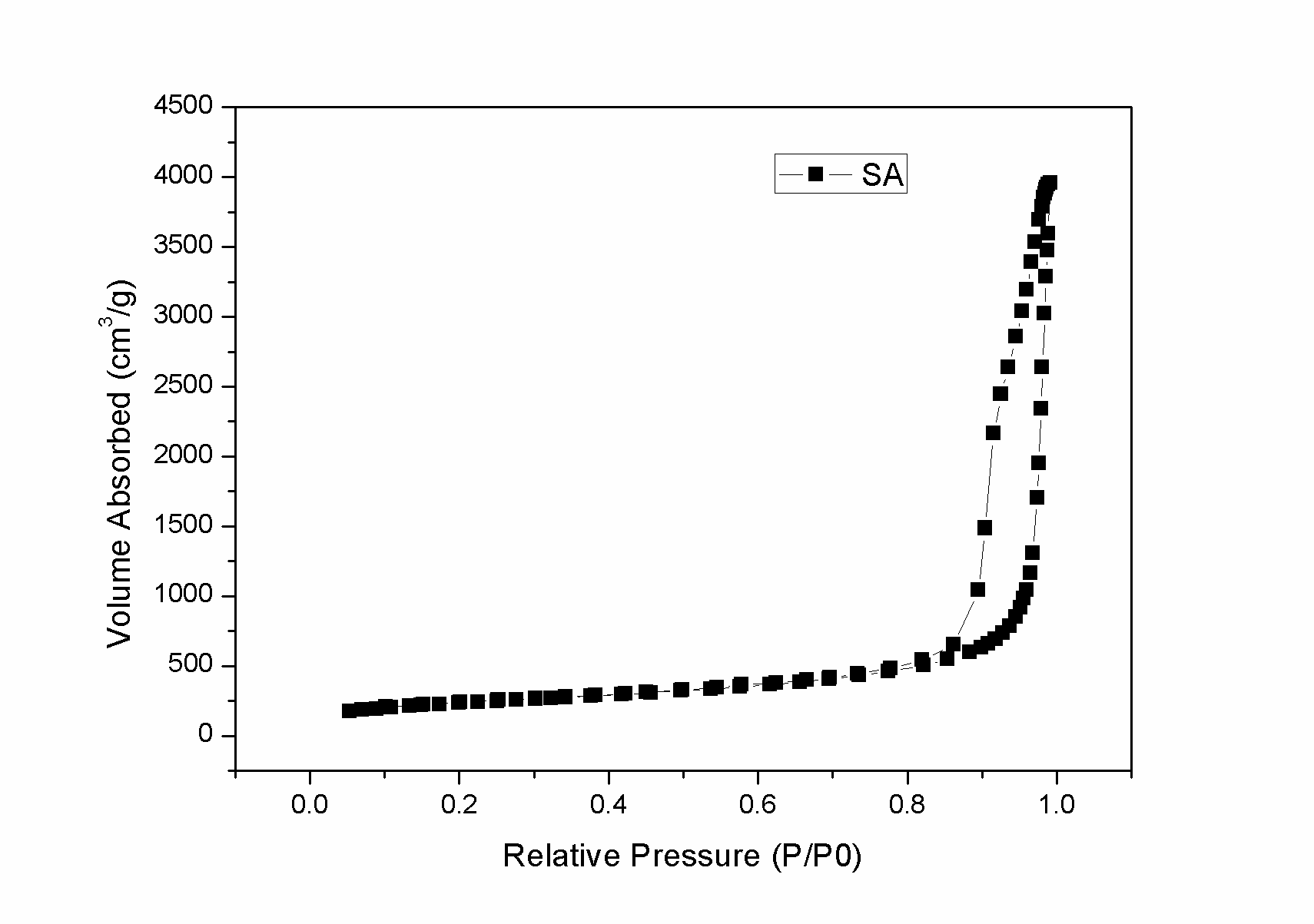

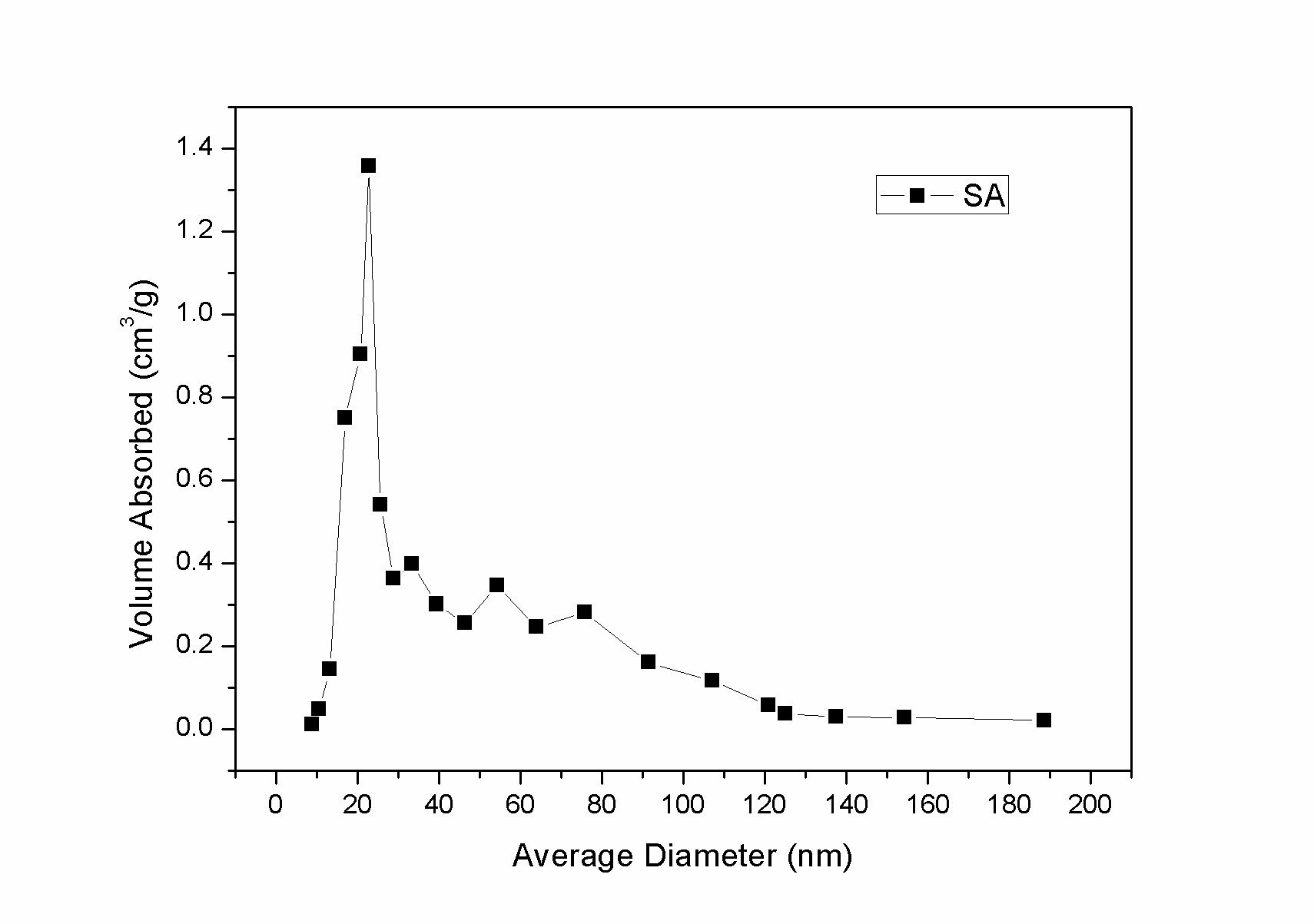


**Figure S2** N_2_ adsorption−desorption isotherms and the corresponding BJH pore size distribution of SA.

**Table S1** BET surface area (S_BET_), pore volume (V_p_) and average pore diameter of SA

| Sample | S_BET_ (m^2^ g^-1^) | V_p_ (cm^3^ g^-1^) | Pore diameter (nm) |
| --- | --- | --- | --- |
| SA | 821.1 | 5.54 | 32.2 |

*
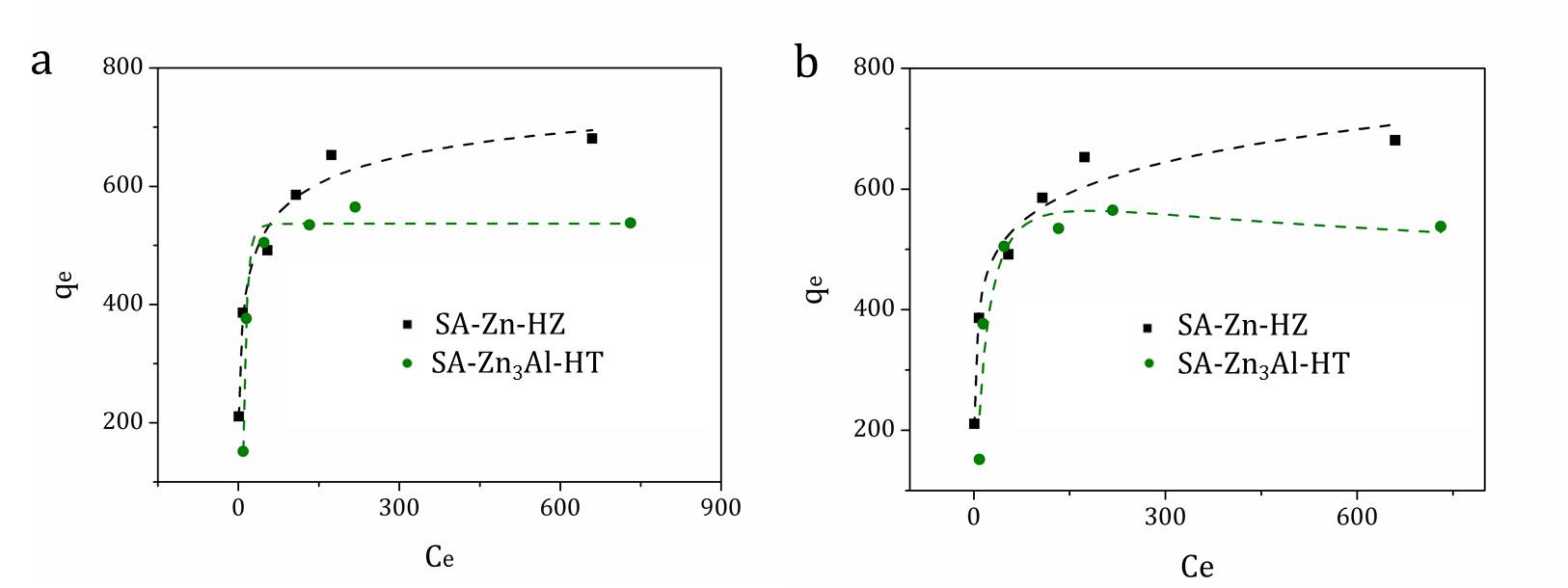
*

**Figure S3** Sips (a) and Redlich-Peterson (b) adsorption isotherm models fitting for Pb(II) adsorption. Initial Pb(II) concentration: 100~1000 ppm; adsorbent dose: 0.5 g·L^-1^; contact time: 24 h, solution pH: 6.0 ± 0.1; temperature: 30^o^C.

**Table S2** The parameters of the Sips and Redlich-Peterson models fitted to the experimental data of Pb(II) adsorption on SA-Zn-HZ and SA-Zn_3_Al-HT.

| adsorbent |  | Sips model parameters | | | |  | Redlich–Peterson model parameters | | | |
| --- | --- | --- | --- | --- | --- | --- | --- | --- | --- | --- |
|  |  | *q*_m_  (mg/g) | K_S_  (L/mmol) | n_S_ | R^2^ |  | *q*_m_  (mg/g) | K_RP_  (L/mg) | n_RP_ | R^2^ |
| SA-Zn-HZ |  | 868.9 | 0.06 | 0.38 | 96.7% |  | 304.8 | 2.29 | 0.88 | 95.3% |
| SA- Zn_3_Al-HT |  | 537.0 | 0.09 | 3.48 | 97.9% |  | 162.9 | 0.05 | 1.09 | 85.7% |

**Figure S4** EDS elemental mapping and the corresponding elemental analysis reports of the SA-Zn-HZ-Pb after the adsorption.

**Figure S5** EDS elemental mapping and the corresponding elemental analysis reports of the SA-Zn_3_Al-HT-Pb after the adsorption.
